# Snow depths’ impact on soil microbial activities and carbon dioxide fluxes from a temperate wetland in Northeast China

**DOI:** 10.1038/s41598-020-65569-x

**Published:** 2020-05-26

**Authors:** Xue Wang, Xueyuan Bai, Liang Ma, Chunguang He, Haibo Jiang, Lianxi Sheng, Wenbo Luo

**Affiliations:** 1 State Environmental Protection Key Laboratory of Wetland Ecology and Vegetation Restoration, School of Environment, Northeast Normal University, Changchun, China 130117; 2 Key Laboratory of Vegetation Ecology, Ministry of Education, Northeast Normal University, Changchun, China 130117

**Keywords:** High-throughput screening, Carbon cycle

## Abstract

Snow depth may have a complex influence on carbon cycling in winter. Here we set up a field experiment to investigate how different snow depths (0 cm, 60 cm, 90 cm) influenced carbon dioxide (CO_2_) in a wetland. The mean ± standard error of CO_2_ emissions under snow addition treatments (60 cm and 90 cm snow depths) were 0.92 ± 0.16 g·cm^−2^·s^−1^ and 0.53 ± 0.16 g·cm^−2^·s^−1^, respectively, compared with snow removal treatment (0 cm snow depth), 0.11 ± 0.05 g·cm^−2^·s^−1^. In general, snow addition increased CO_2_ fluxes significantly. As snow depths increased, microbial biomass carbon (MBC) and bacterial diversities increased drastically. More important, the community of bacteria differed under different treatments. *Firmicutes*, which can resist dehydration and extremely low temperatures, was widely distributed in the snow removal treatment, where it sustained soil biochemical processes. Overall, our study indicates that snow cover counteracts the negative effects on soil microbial activities caused by low temperatures and could play a critical role in winter carbon cycling in wetlands.

## Introduction

As a result of global warming, precipitation (including snow) has changed significantly during the past several decades, and permafrost in high latitude areas has begun to melt^1,2^. Soil organic carbon exposed to the air was decomposed by microorganisms and emitted into the atmosphere, which may aggravate global warming^3^. Therefore, there has been a large amount of research interest on carbon cycling in the context of climate change^4,5^, especially CO_2_. Wetlands in particular are generally considered as a large soil organic carbon pool^6^. Although northern hemisphere wetlands cover only 3% of the earth’s land area, they store 400–600 Gt of C, accounting for 1/4 to 1/3 of global soil organic carbon^7–9^. So it is very important to develop studies of carbon cycling in these regions. However, most of the studies were set up in growing season, but few in winter^10^. Several studies have shown that carbon emissions still occur in winter in northern wetlands, and these emissions also play an important role in global carbon cycling^11–13^. More than 3–40% of carbon accumulated through photosynthesis in the growing season will release through winter respiration^14^, accounting for about 4.4–21.6% of annual carbon releases^11–13,15^. However, this type of research has not yet been performed sufficiently, due to the difficulties of field measurements in winter, and still requires further discussion^10,16^.

Snow is the major variable in northern wetlands in winter, impacting CO_2_ fluxes in wetlands. For example, Blankinship and Hart (2012) found decreased snow depth could cause a 35% reduction in CO_2_ fluxes^17^. Li *et al*. (2016) also found that increased snow depth could increase the soil-atmosphere CO_2_ fluxes^18^. Since snow cover isolates soil from cold air, it usually generates a soil microclimate for carbon emissions^19,20^. However, there are still few experiments which have mainly studied how different snow depths impact on winter CO_2_ fluxes^20–25^.

Many researchers found that soil enzymes were catalysts in soil, participating in almost all soil biochemical processes, and they have a major influence on the carbon cycling of terrestrial ecosystems^26,27^. Among these, soil invertase belongs to hydrolase, which plays an important role in the process of carbon cycling. Under the condition of snow cover, soil temperature usually remains at approximately 0 °C and sometimes even higher, which might be warm enough to maintain soil biotic activities during the winter period^28^. Thus, higher soil temperatures may enhance soil enzyme activities to accelerate soil decomposition processes during winter time, which might be one of the most important ways that snow depths impact CO_2_ fluxes in winter^29–31^. However, to our knowledge, few studies have focused on this.

Several studies have reported that soil microbial biomass is even greater in the winter time than that in summer season in some regions^32,33^. More important, microorganisms are capable of maintaining catabolic (CO_2_ production) and anabolic (biomass synthesis) processes under sub-zero temperatures^30,34,35^. For example, Brooks *et al*. (1996) found that deep and continuous snow cover significantly increased microbial biomass carbon (MBC) and microbial decomposition rates^22^. All above studies indicated that CO_2_ emitted by soil microbial respiration in winter is an important part of the annual carbon budget^36,37^. Thus, there is an urgent need to explore how microbial community composition responds to snow cover, which is important for better understanding the mechanisms of subnivean CO_2_ emissions^38,39^. However, traditional methods such as phospholipid fatty acid (PLFA) analysis or polymerase chain reaction-denaturing gradient gel electrophoresis (PCR-DGGE) provided limited phylogenetic or taxonomic resolution of the influence of snowpack on microbial communities^40^. By comparison, high-throughput sequencing technology can clearly describe the soil microbial diversities and composition. For example, Liptzin *et al*. (2015) reported that in the Niwot area of Colorado, winter soil bacteria mainly included *Cytophaga* and *Flexibacter*, but the dominant microorganism under snowpack was SMF (snow mold fungi)^41^. Whether or not this conclusion is suitable for other regions has yet needed to be confirmed. Therefore, the mechanisms of which snow depth influences CO_2_ fluxes were insufficiently clear, especially regarding the microorganisms involved.

Here, we conducted a field experiment using snow gradient manipulation in Jinchuan wetland located in the west of Jinchuan Town, Huinan County, Jilin Province, China, which is known as the most concentrated group of crater lake wetlands, during the winter period from 2016 to 2017. Firstly, we tried to clarify how different snow depths influence CO_2_ emissions; Secondly, we attempted to explore the influence mechanisms of snow depth on CO_2_ fluxes, and whether soil invertase could make sense of subnivean CO_2_ emissions; Thirdly, whether MBC and bacterial composition might also influence subnivean CO_2_ emissions.

## Materials and Methods

### Site description and experimental design

**Figure 1:**
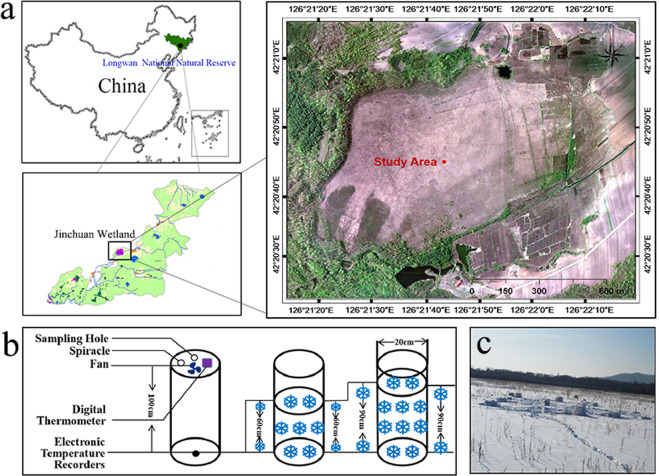
Map of the study area located in Longwan National Natural Reserve in the Jinchuan wetland in China (**a**)^42^; Diagram of the experimental device (**b**: Schematic diagram of chamber; **c**: Actual layout of device).

The experiment was developed in a Jinchuan wetland located in Longwan National Natural Reserve, Jilin Province, China (42°20′56″N, 126°22′51″E) (Fig. 1a), as described by Shi *et al*.^42^. It has a north temperate monsoon climate, with a long winter, from October to May. The average annual temperature and precipitation are approximately 3 °C and 1053.9 mm, respectively^43^. Previous investigations have shown that Jinchuan wetland has a constant peat accumulation rate of approximately 1 mm/a, providing a carbon stock of approximately 4,599,300 m^3^^43.44^. The wetland has a thick layer of peat, with a maximum thickness of 10 m.

Previous studies by our group have included an investigation of the background conditions of the study area, including hydrological conditions, elevation, vegetation and other environmental factors. The average snow depth was approximately 30-70 cm. According to these data, a region of 300 m^2^ were selected in which most of the biotic and abiotic properties were relatively homogeneous to develop our experiment. The snow gradient manipulation field study was conducted using a completely random design. Three snow depth treatments were included in this study, including 0 cm, 60 cm, and 90 cm, with five replicates of each snow depth randomly distributed in the area. There were 15 plots, each of them measured 1.5 m × 1.5 m and was encircled by four iron rods fixed in the soil. Closed static-chambers were fixed by connecting with the iron rods. The plots were separated by a buffer strip (3 ± 2 m) to control edge effects^31^ (Fig. 1c).

Electronic temperature recorders (DS1921G-F5, China, Shanghai Wassersen Electronic Technology Co. LTD.) were installed at 30 cm soil depth to record the soil temperature of each snow depth treatment. Also a 3-hour interval was set to measure the soil temperature continuously during the entire experimental period.

### CO_2_ flux measurements

CO_2_ fluxes were measured by closed static-chamber technique and subsequent gas chromatographic analysis. Chambers were made of polymethyl methacrylate, with a diameter of 20 cm and a height of 100 cm. Every chamber was equipped with a spiracle, and a sampling hole, connected with silicone tubes, which were sealed by water stoppers. In addition, to record the real-time temperature in the chambers, ST-1A digital thermometers (−50 °C– + 80 °C, BT-12M 1.3AT, Digital thermometers) were installed in the chambers. Fans (12 v, 7 cm × 7 cm) were powered by accumulators, and they were necessary to keep gases homogeneous in the chambers (Fig. 1b).

We used shovels to manipulate snow depths. As for 0 cm snow depth treatment, a small amount of snow was left on the ground to keep the albedo homogeneous among the plots^45^. The treatments with snow depths of 60 cm and 90 cm were controlled by chambers. To keep the snow depths equal in and outside the chambers, nylon mesh and iron wire were used to delineate the plots, which were then filled in with different quantities of snow. Moreover, as soon as snow events occurred, snow depth modulation was performed immediately in order to maintain the snow depth within appropriate limits.

Collection of gasses took place when melting events occurred. Gases were collected into evacuated aluminum plastic composite membrane sampling bags with a sampling pump fortnightly. Collection always occurred after the fans had been running for 5 min. All the gases were collected at the interval of 15 min, namely 0, 15, 30, and 45 min, and then we took them back to the laboratory. Gases samples were stored at room temperature before gas chromatographic analysis. Fluxes were calculated by the change rate of gas concentration as follows:

$$F=(\rho \cdot V\cdot P\cdot {T}_{0}/A\cdot {P}_{0}\cdot T)\cdot (dCt/dt)$$

where *F* (g/cm^2^·s) refers to gas flux, *ρ* (g/ml) is the density of gas under standard conditions, *V* (cm^3^) is the volume of air in the chamber, *A* (cm^2^) is the covered area of the chamber, *P* (Pa) is the pressure of the sampling point, *P*_*0*_ (Pa) is the absolute air pressure under standard conditions, *T*_*0*_ (K) is the absolute air temperature under standard conditions, *T* (K) is the absolute temperature in the chamber when sampling, and *dCt*/*dt* is the change rate of gas concentration.

### Collection of soil samples

We collected 0-30 cm soil samples, and 2 soil cores in each plot, then mixed them together, subsequently put them in marked sealed bags and took them back to the laboratory. After quick removal of gravel, main roots and other debris in soil, some of the soil samples were stored in the refrigerator at 4 °C, and were used for measuring MBC. Some of the soil samples were air-dried for measuring invertase activities. Other soil samples were stored in the Ultra-low temperature freezer at -80°C for high throughput sequencing.

### MBC and soil invertase measurements

MBC was measured via the method called “chloroform fumigation extraction”^46^, “3,5-dinitrosalicylic acid colorimetry” was used to measure invertase activities^47–49^.

### Soil high-throughput sequencing technology

The experiment included three snow depth treatments (0, 60 cm, 90 cm), with three repetitions for every treatment, marked 1, 2, 3, respectively. Therefore, the samples were marked as 0-1, 0-2, 0-3, 60-1, 60-2, 60-3, 90-1, 90-2, 90-3. After DNA extraction, genomic DNA was detected by 1% agarose gel electrophoresis. The next step was to conduct PCR amplification, the V3-V4 hypervariable region of bacterial 16S rRNA gene were amplified with the primers 338F (ACTCCTACGGGAGGCAGCAG) and 806R (GGACTACHVGGGTWTCTAAT)^50^. For each soil sample, 10-digit barcode sequence was added to the 5’end of the forward and reverse primers (provided by Allwegene Company, Beijing). The PCR was carried out on a Mastercycler Gradient (Eppendorf, Germany) using 25 μl reaction volumes, containing 12.5 μl 2 × Taq PCR MasterMix, 3 μl BSA (2 ng/μl), 2 Primer (5 uM), 2 μl template DNA, and 5.5 μl dd H_2_O. Cycling parameters were 95 °C for 5 min, followed by 32 cycles of 95 °C for 45 s, 55 °C for 50 s and 72 °C for 45 s with a final extension at 72 °C for 10 min. The PCR products were purified using a QIAquick Gel Extraction Kit (QIAGEN, Germany), quantified using Real Time PCR, and sequenced at Allwegene Company, Beijing. Then PCR products were detected and quantified by the “blue fluorescence quantitative system QuantiFluor™-ST (Promega Company)”. Then, a MiSeq library was constructed and sequenced on the machine. Raw sequencing reads obtained in this study have been deposited into the NCBI Sequence Read Archive (SRA) database with the accession number of SRP250149.

### Statistical analyses

All of the statistical analyses were performed using IBM SPSS software (Version 19.0) and graphed by SigmaPlot 12.5. All potential factors of influence (MBC and soil invertase activities) on CO_2_ fluxes under different snow depths were assessed by one-way anova analysis (with interactions by Duncan test). Daily average soil temperature was computed by taking the mean values measured at an interval of 3 hours.

Operational taxonomic units (OTUs) were formed by cluster analysis, and then bioinformatics and phylogenetic analyses were conducted with a similarity of 97%. The Kruskal-Wallis test was used to evaluate the difference of soil bacteria under different snow depths. Principal coordinates analysis (PCoA) was performed using R to examine microbial community structural variances between different snow depth treatments based on Weighted-UniFrac distance. The P value was calculated by permanova via python. Alpha diversity indices of bacteria (Chao1 richness) were calculated through the number of soil bacteria species (OTUs) and 16S rDNA sequence number (Reads). The formula of Chao1 richness (Bacterial richness index) is:

1 $${\rm{Schao}}1={\rm{Sobs}}+{\rm{n1}}({\rm{n1}}-1)/2({\rm{n2}}+1)$$

where Schao1 is the estimated number of OTUs, Sobs is the number of observed OTUs, n1 is the number of OTUs with only one sequence, and n2 is the number of OTUs with only two sequences. The data were analyzed with QIIME^51^. Based on the unweighted UniFrac distance matrix, UPGMA (Unweighted Pair Group Method with Arithmetic Mean) was used for cluster and tree construction, and the results of clustering were integrated with the relative abundance of species at the phylum level.

## Results

### CO_2_ fluxes

**Figure 2:**
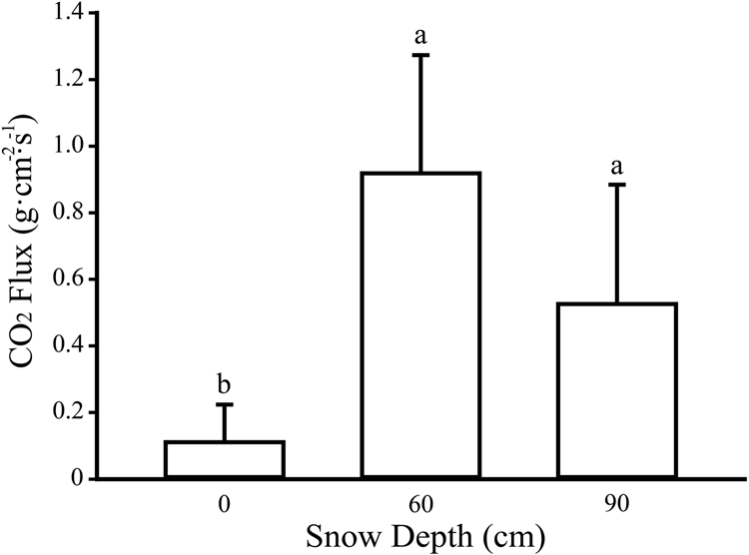
CO_2_ fluxes under different snow depths.

Soil CO_2_ fluxes varied under different snow depth treatments. The mean CO_2_ flux under snow removal treatment (0 cm) was 0.11 ± 0.05 g·cm^−2^·s^−1^, while CO_2_ fluxes under 60 cm and 90 cm snow depth treatments were 0.92 ± 0.16 g·cm^−2^·s^−1^ and 0.53 ± 0.16 g·cm^−2^·s^−1^, respectively. It indicated that 60 cm and 90 cm snow depth treatments showed significantly increased CO_2_ flux compared with 0 cm snow depth (Fig. 2, P = 0.004), but there was no significant difference between snow cover treatments.

### Soil temperature and soil invertase activities

**Figure 3:**
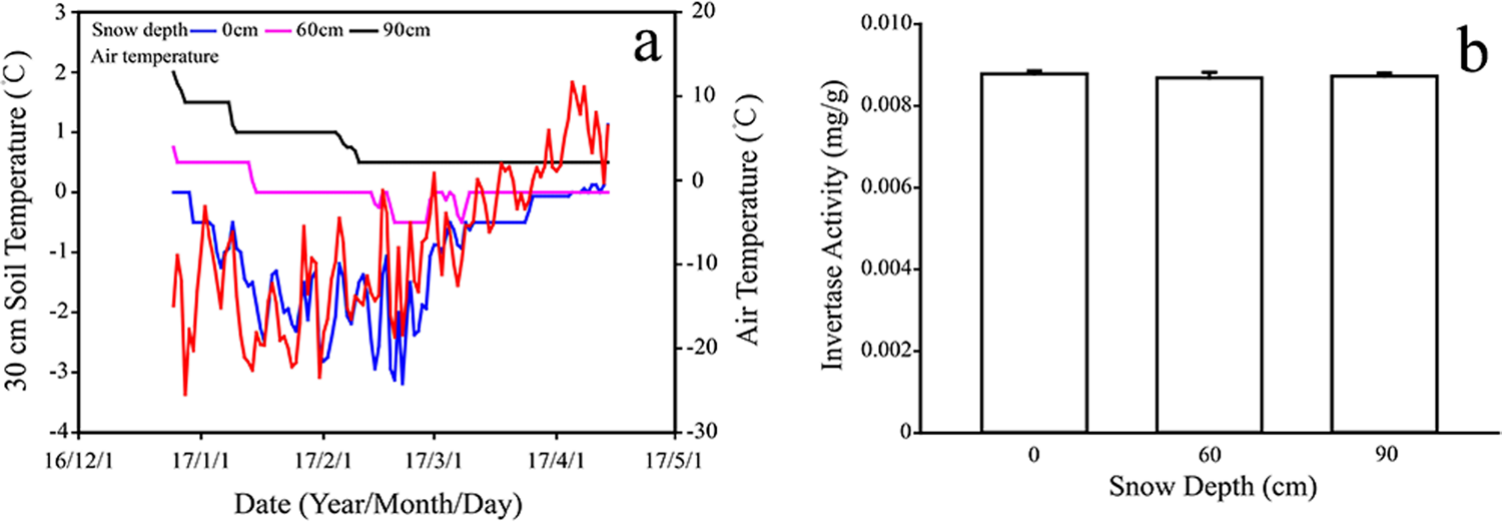
(**a**) Values are daily average soil temperature in 30 cm soil depth with different snow depths of 0 cm, 60 cm and 90 cm range from December 2016 to April 2017; Soil invertase activities (**b**) under different snow depths.

As is known to all, soil enzyme activities are closely related to soil temperature. In this study soil temperature increased with the increased snow depth. It tended to be more stable and was barely affected by air temperature with thicker snow cover (Fig. 3a). However, this snow manipulation experiment did not significantly affect the soil invertase activities (Fig. 3b).

### Soil microorganisms

**Figure 4:**
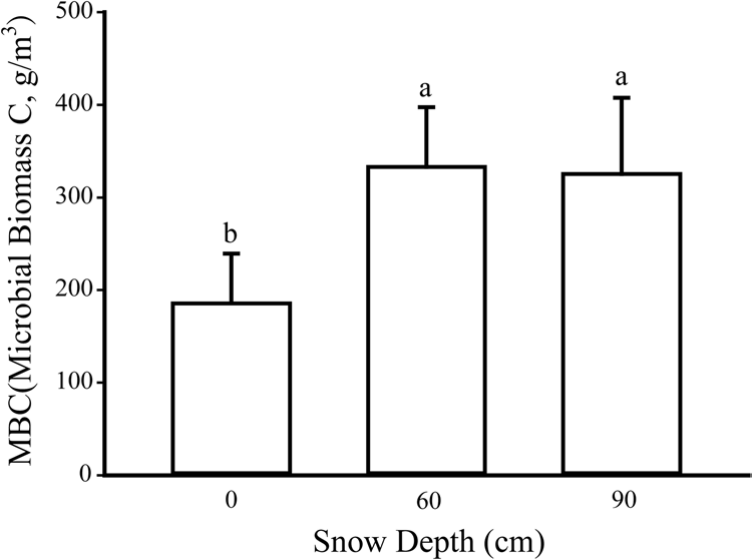
Values are MBC under different snow depths.

**Figure 5:**
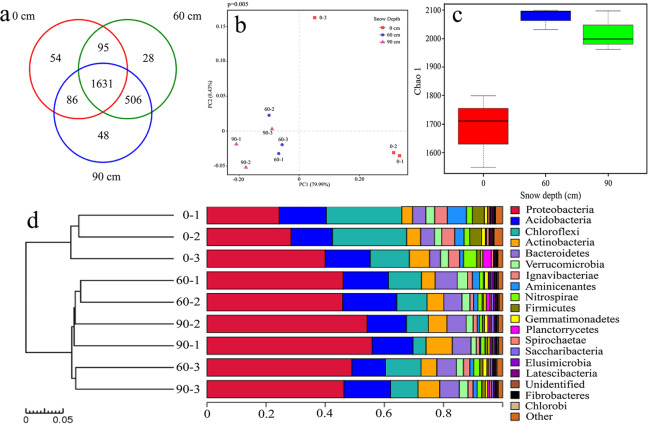
Soil bacterial community structure and diversities under different snow depth treatments: (**a**) shows a Venn diagram in which the overlapping parts are OTUs shared by different treatments; (**b**) shows PCoA based on Weighted-UniFrac distance; (**c**) is a box diagram of alpha diversity index (Chao1 index) for different snow depth groups. (**d**) is a cluster histogram of samples. On the left is the hierarchical cluster analysis, and the community structure histogram is on the right.

MBC showed the same tendency as CO_2_ fluxes which also increased markedly under snow cover (Fig. 4, p < 0.001). The concentrations of MBC were 185.52 ± 53.83 g·m^−3^, 332.95 ± 64.50 g·m^−3^ and 325.36 ± 82.30 g·m^−3^ under the treatments of 0 cm, 60 cm and 90 cm snow depths, respectively. High throughput sequencing results are as follows, valid data is shown in Table 1 (raw-tags gained after splicing of the filtered low-quality fastq data, and clean-tags are the results of further removing chimera and short sequence from splicing results). The distribution of OTUs under different snow depths is indicated by the Venn diagram (Fig. 5a). In total, this study generated 2448 OTUs, including 1631 OTUs that were present in all snow depths. The results of PCoA indicated that microbial community varied significantly (P = 0.005) between different treatments (Fig. 5b).

**Table 1:** Splicing result statistics.

Name of samples	Raw tags	Clean tags
0–1	33995	33291
0–2	41582	40472
0–3	39322	37985
60–1	42336	40526
60–2	45827	43897
60–3	34138	33274
90–1	39431	37519
90–2	43960	42064
90–3	45749	44140

The alpha diversity index was used to compare microbial community diversity among different snow cover groups; snow cover significantly increased the alpha diversity indices of bacteria (Fig. 5c, Chao1 richness, P = 0.003). The dominant bacteria in Jinchuan wetland were *Proteobacteria*, *Acidobacteria*, *Chloroflexi*, *Actinobacteria*, and *Bacteroidetes* (Fig. 5d). The distribution of bacteria showed totally different in different snow cover groups. *Proteobacteria* increased significantly with increasing snow depth (P = 0.039). *Chloroflexi* (P = 0.027), *Firmicutes*, and *Nitrospirae* were relatively abundant in 0 cm snow depth (Table 2). At the genus level, *Pseudolabrys*, *Candidatus_*Solibacter, *Bradyrhizobium*, *Candidatus_*Koribacter, *Bryobacter*, *Candidatus* Competibacter, and *Geobacter* took an important place in winter soil bacteria; *Pseudolabrys*, *Bradyrhizobium, Bryobacter*, and *Mucilaginibacter* increased significantly with increasing snow depth (P = 0.039, P = 0.027, P = 0.027, P = 0.027) (Table 2).

**Table 2:** Summary of Kruskal-Wallis rank-sum test results for the top 10 dominant phyla and genera in different snow depth treatments.

	Name	0 cm-mean	60 cm-mean	90 cm-mean	P-value
Phyla	Proteobacteria	0.31	0.47	0.52	0.04^*^
	Acidobacteria	0.15	0.15	0.14	0.73
	Chloroflexi	0.21	0.11	0.07	0.03^*^
	Actinobacteria	0.05	0.05	0.08	0.12
	Bacteroidetes	0.04	0.07	0.06	0.07
	Verrucomicrobia	0.03	0.03	0.02	0.43
	Ignavibacteriae	0.04	0.02	0.01	0.06
	Aminicenantes	0.04	0.02	0.01	0.15
	Nitrospirae	0.03	0.02	0.01	0.11
	Firmicutes	0.03	0.01	0.01	0.05
Genera	Unidentified	0.72	0.64	0.59	0.11
	Pseudolabrys	0.01	0.03	0.04	0.04^*^
	*Candidatus*_Solibacter	0.02	0.02	0.03	0.19
	Bradyrhizobium	0.01	0.02	0.03	0.03^*^
	*Candidatus*_Koribacter	0.01	0.02	0.01	0.39
	Bryobacter	0.01	0.01	0.02	0.03^*^
	*Candidatus* Competibacter	0.02	0.01	0.01	0.84
	Geobacter	0.01	0.01	0.01	0.96
	Rhodomicrobium	0.01	0.01	0.01	0.67
	Duganella	0.01	0.01	0.01	0.73

*P < 0.05.

## Discussion

Many researches have studied the influences of snow depth on CO_2_ fluxes during the winter period^20,41^. They found that snow removal would cause a reduction of CO_2_ fluxes, whereas snow addition would increase CO_2_ fluxes (Table 3). In this study, we also found similar outcomes and also determined that snow addition will cause higher CO_2_ emissions during the winter period. However, the relationship between snow depth and CO_2_ fluxes did not show a totally positive correlation, as value under the treatment of 60 cm was slightly higher than that in 90 cm (Fig. 2). Based on our results, CO_2_ flux may be related to the following three points: Firstly, it may be related to the soil temperature of the optimum active layer in the wetland, which is usually at 30–40 cm. Secondly, the fluctuation of temperature may have a “stimulating” effect, the results of Fig. 3a show that soil temperatures under 60 cm snow depth fluctuated more than those under 90 cm. Neilson *et al*. (2001) studied forest soil and confirmed our speculations, indicating that the greater the fluctuation in soil temperature, the greater the CO_2_ fluxes^52^. Thirdly, the pulse intensities are different under different snow depths, suggesting some of the gases may be trapped in snow and ice. Therefore, further research is needed to investigate the other causes.

**Table 3:** A comparison of snow manipulation experiments and carbon dioxide fluxes with different snow depths estimated from different ecosystems at the similar latitude.

Geographical location	Ecosystems	Snow manipulation technique	Natural snow depth (m)	Direction of snow manipulation	Manipulated snow depth (m)	Measurement method	Time	Impact on CO_2_ flux	Citation
43°56′N, 71°45′W	Forest (birch)	Shovel	0.5~1.5	Removal	0.31, 0.23	Chamber	Two winters (1997/1998 and 1998/1999)	Reduction	[70]Groffman *et al*., 2006
43°56′N, 71°45′W	Forest (maple)	Shovel	0.5~1.5	Removal	0.31, 0.23	Chamber	Two winters (1997/1998 and 1998/1999)	Reduction	[70]Groffman *et al*., 2006
40°03′N, 105°35′W	Alpine tundra	Snow Fence	Shallow snowpack sites(Maximum depth <1); Deep snowpack Sites (Maximum depth 1.5–2)	Addition	Deepened snowpack	Fick’s diffusion model	One winter (1993/1994)	Increment	[29]Brooks *et al*., 1997
48°02′–48°12′ N, 128°58′–129°15′ E	Pine forest	Fence	0.33 ± 0.05 ~ 0.42 ± 0.04	Removal	0, 0.1, 0.2, 0.3	Li-8100 Automated Soil CO_2_ Flux System	One winter (2013/2014)	Reduction of snowpack-Decreased	[10]Liu *et al*., 2016
41°18′47″N, 105°59′46″E 41°15′6″N, 105°26′6″E	Sagebrush steppe	Fence	—	—	0~2	Chamber	One winter (2012/2013)	Increasing of snowpack-Increased	[71]Tucker, *et al*., 2016
42°20′56″N, 126°22′51″E	Wetland	Shovel Chamber	0.3–0.7	Addition	0, 0.6, 0.9	Chamber	One winter (2016/2017)	Increment	This study
				Removal				Reduction	

Invertase activity is related to an important nutrient for soil microbes (glucose)^53^, which is also vital for carbon emissions. However, invertase activities showed homogenous among different snow depths, which might prove the effects of snow cover on CO_2_ fluxes may not result from the shortage of nutrients (eg. sucrose) in soil. Additionally, soil enzyme activities are also driven by soil temperature, which was also effected by snow cover. Through our observation of the whole winter, soil temperature maintained at a constant temperature of 0°C-2°C. Previous studies have shown that snow cover can isolate soil from cold air and temper the atmospheric temperature changes’ influence on soil^54^. It could also increase soil moisture around the soil particles. As a result, it is beneficial to soil microbial activities^18,41^, thus ensuring the activity of soil enzymes^55,56^. In summary, this answered the second question, clarifying that invertase activities can ensure subnivean CO_2_ emissions in winter, but it might not be the major reason of the different subnivean CO_2_ emissions.

Studies indicated that MBC is also pivotal for CO_2_ emissions. Brooks and Williams (2010) found deeper and continuous snow cover could accelerate decomposition rates and lead to a higher MBC^57^. Similarly, our study showed that snow addition significantly increased MBC. Moreover, Sylvia *et al*. (2005) indicated that higher snow depths increased litter biomass, which increases labile carbon input^58^. This may explain the increase of MBC under snow addition. More important, MBC is one of the most active component of soil organic carbon and can directly participate in soil biochemical processes^59^. As a result, increased MBC might increase carbon output (CO_2_), thus affirmatively answering our third question as to whether MBC also impacts subnivean CO_2_ emissions.

Soil bacterial composition and richness also differs among different snow depths in our study. At the phylum level, major taxa in our study area were similar with those found in other studies^60^. At the genus level, Liptzin *et al*. (2015) reported that the major soil bacteria in Niwot Ridge of the Colorado Rocky Mountains were *Cytophaga* and *Flexibacter* in winter^41^. By contrast, our research has drawn different conclusions. *Flexibacter* were not observed in our study, and *Cytophaga* was also not a major taxon. In this study, *Pseudolabrys*, *Candidatus_*Solibacter, *Bradyrhizobium*, *Candidatus_*Koribacter, *Bryobacter*, *Candidatus_*Competibacter and *Geobacter* occupied an important position. It was considered that this dissimilarity was caused by differences in soil background or regional environment. Also, Dong *et al*. (2017) reported that higher snow depth caused a significant increase of bacterial richness and diversity, and played an important role in shaping microbial communities^61^. Additionally, Gao *et al*. (2020) found 16S rRNA gene abundance was positively correlated with soil CO_2_ emissions^62^. Microbial abundances and elevated temperature may increase the metabolic activity of microbes^63^ and decomposition of organics^64^. Similarly, this study reported that higher richness of bacterium appeared in subnivean soil, suggesting that this condition may provide more niches for microbial taxon. Nevertheless, how these dominant bacteria impact subnivean CO_2_ emissions still needs to be further investigated. Different bacteria also responded to snow depth with different sensitivities even in the same area. Several studies have reported most of the taxa found in cold winter conditions can correspond to either microorganisms with specific adaptive strategies like spore formation, or to opportunists^65,66^. Through our study, *Proteobacteria* increased significantly with increasing snow depth, whereas *Firmicutes* decreased. Ma (2013) conducted a study on Antarctic tundra and reported that soil carbon showed a negative correlation with the relative abundance of *Proteobacteria*^67^. It may correlate with the enhancement of other modalities of carbon, such as CO_2_. Moreover, many kinds of *Firmicutes* can resist dehydration and extreme conditions through producing spores at low temperature to maintain the soil biochemical processes. Additonally, previous study proved that the distinct presence of the phylum *Chloroflexi* at the temperature-reducing condition^68^, possibly due to the symbiosis with other bacteria by utilising cellular compounds and metabolites derived from dead biomass^69^.

The impact of snow depth on carbon dioxide emissions in winter is complex and worthy of further study in the context of global climate change. The insulating function of snow cover ensures liquid water around soil particles by increasing soil temperature. Also the elevated subnivean temperatures enhance the metabolic and decomposition activity of microorganisms, thus enhancing the carbon emissions.

## Conclusion

The effects of altered snow depth on wetland greenhouse gases were complex. Thicker snow depth could accelerate the process of carbon cycling. Our research also revealed the impacts of snow depth on CO_2_ fluxes were mainly related to soil microbial activities (soil invertase activities, MBC, bacterial composition and bacterial richness). Higher snow depth increased microbial diversities, which had played a positive effect on carbon cycling. This field experiment could indicate the flux of carbon emissions under various snow depth and climate change scenarios. Additionally, the balance of the carbon budget in wetland ecosystem were associated with many vital factors, especially microbiological changes in freeze-thaw cycle stage. Therefore, more long-term studies should be developed to explore the impacts of soil microorganisms during this period.
