# Author Correction: Snow depths’ impact on soil microbial activities and carbon dioxide fluxes from a temperate wetland in Northeast China

**DOI:** 10.1038/s41598-020-69047-2

**Published:** 2020-07-15

**Authors:** Xue Wang, Xueyuan Bai, Liang Ma, Chunguang He, Haibo Jiang, Lianxi Sheng, Wenbo Luo

**Affiliations:** 1 State Environmental Protection Key Laboratory of Wetland Ecology and Vegetation Restoration, School of Environment, Northeast Normal University, Changchun, 130117 China; 2 Key Laboratory of Vegetation Ecology, Ministry of Education, Northeast Normal University, Changchun, 130117 China

Correction to:* Scientific Reports* 10.1038/s41598-020-65569-x, published online 26 May 2020

This Article contains an error in Figure 3, where the label for ‘Air Temperature’ is missing. The correct Figure 3 appears below as Figure 1.

**Figure 1:**
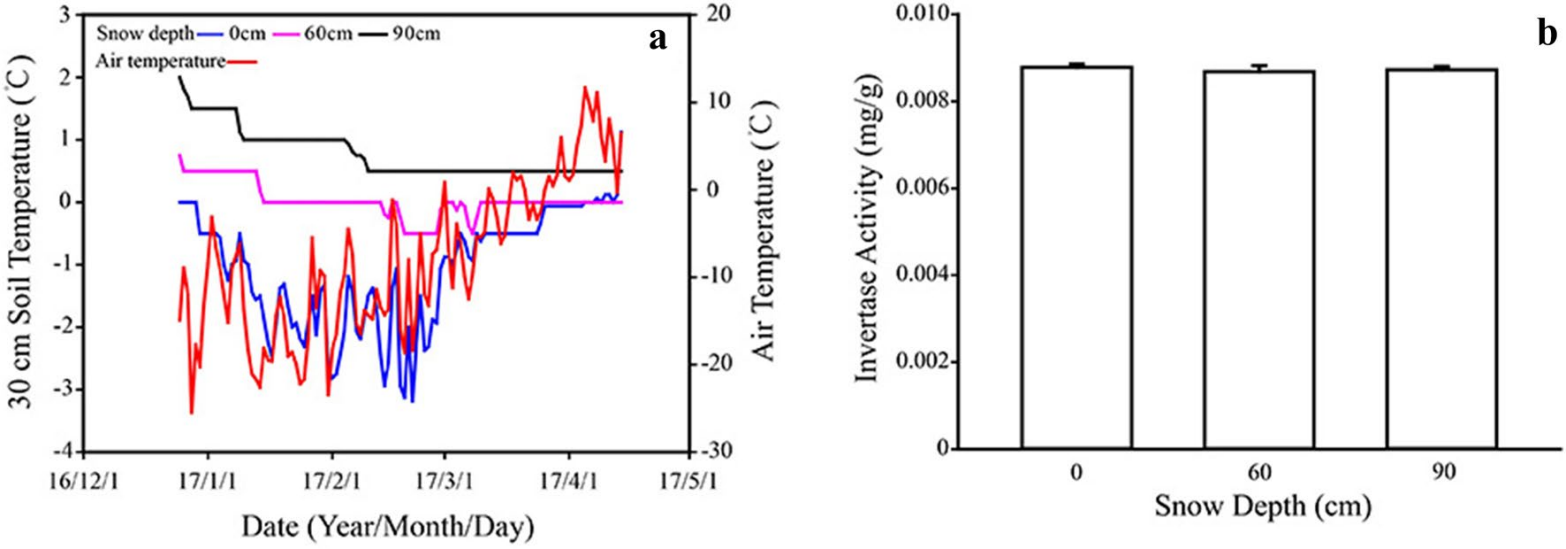
Figure 1 **(a)** Values are daily average soil temperature in 30 cm soil depth with different snow depths of 0 cm, 60 cm and 90 cm range from December 2016 to April 2017; Soil invertase activities** (b)** under different snow depths.

